# Varying clinical presentations of nutcracker syndrome: a case report

**DOI:** 10.1186/s13256-025-05156-8

**Published:** 2025-04-01

**Authors:** K. K. Athish, N. Prasanna Kumar, Shobhana Nayak-Rao

**Affiliations:** Department of Nephrology, Sri Devaraj Urs Academy of Higher Education and Research, Tamaka, Kolar, 563103 Karnataka India

**Keywords:** Hematuria, Left renal vein, Compression, Flank pain, Surgery

## Abstract

**Introduction:**

The term nutcracker syndrome was initially established in 1972 to refer to the compressive effects on the left renal vein due to a narrow angle between the abdominal aorta and the superior mesenteric artery. Although the precise prevalence of nutcracker syndrome remains unknown, an incidence of up to 4% has been described in patients presenting with hematuria. The peak age of incidence is between 10 and 30 years, and it is equally prevalent among both genders, though earlier studies showed a predominantly female preponderance. About 70–80% of patients present initially with hematuria, though left flank pain and proteinuria may also be presenting symptoms. A high index of suspicion and appropriate imaging often helps in aiding diagnosis.

**Case presentation:**

In this case report, we present here two South Asian young females aged 23 and 30 years old. They presented with gross painless hematuria of a duration of 2 years (Case 1) and nonspecific symptoms of abdominal pain (Case 2), and they were diagnosed with nutcracker syndrome on investigation. Both patients were diagnosed by computed tomography angiography with defined aortomesenteric angles diagnostic of nutcracker syndrome. Patient 1 underwent saphenous venous bypass grafting and repositioning of left renal vein with symptomatic improvement, while patient 2 was managed conservatively and continues to be on close follow-up. In addition, we present a brief review of this syndrome so that better insight is obtained regarding diagnosis and management.

**Conclusion:**

The diagnosis of nutcracker syndrome needs to be considered in patients who present with unexplained hematuria or proteinuria. Diagnosis by appropriate imaging studies is necessary, and treatment is dictated by the severity of symptoms. Surgical therapy remains the front-line treatment; however, endovascular techniques are becoming favored owing to advancements in stent technology.

## Introduction

The term nutcracker syndrome (NCS) was initially established in 1972 [1] to refer to the compressive effects on the left renal vein (LRV) due to a narrow angle between the abdominal aorta and the superior mesenteric artery (SMA). This is referred to as the aortomesenteric angle (AM), and an AM angle of < 35–39° is generally considered as one of the criteria for the diagnosis on imaging. Although the precise prevalence of NCS remains unknown, an incidence of up to 4% has been described in patients presenting with hematuria [2]. The peak age of incidence is between 10 and 30 years. It is equally prevalent among both genders, though earlier studies showed a predominantly female preponderance [3, 4]. About 70–80% of patients initially present with hematuria; however, left flank pain and proteinuria may also be presenting symptoms. A high index of suspicion and appropriate imaging often aid in diagnosis. We present here two cases of young females who presented with gross painless hematuria of a duration of 2 years (Case 1) and nonspecific symptoms of abdominal pain (Case 2). They were diagnosed with nutcracker syndrome on investigation. Nutcracker syndrome is an uncommon diagnosis and not frequently encountered in nephrology clinical practice; therefore, we present a brief review of this syndrome so that better insight is obtained regarding diagnosis and management.

## Case presentation

### Case 1

**Fig. 1:**
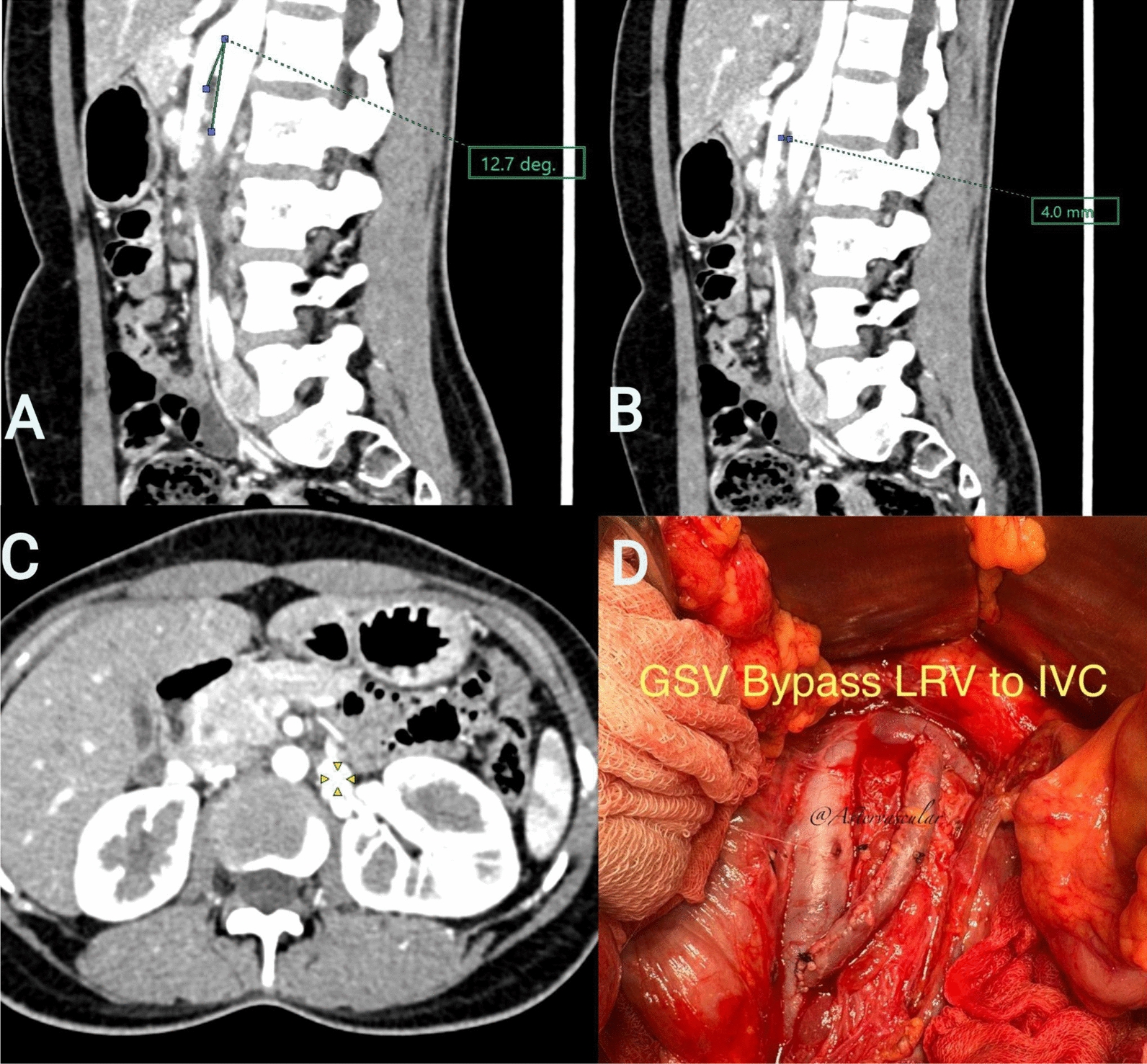
**A** Computed tomography angiogram sagittal section showing aortomesenteric angle of 12.7° with minimal dilatation of gonadal vein; **B** computed tomography angiogram sagittal section showing aortomesenteric distance of 4 mm; **C** computed tomography angiogram axial section showing dilated renal vein (arrows); **D** intraoperative image showing left renal vein bypass grafting was done using the left greater saphenous vein

A 23-year-old South Asian female individual presented to the nephrology clinic with a history of persistent painless gross hematuria that had started about 3 years earlier. She had noticed intermittent reddish-colored urine about 3 years back, but this had become more severe and had been more persistent for the last 2 years. The hematuria was painless and not associated with any systemic symptoms. There was no history of weight loss, fever, or joint pains. Her menstrual history was normal, and she denied a history of menorrhagia. She had a history of syncopal episodes, which were investigated; computed tomography (CT) brain and electrocardiography (ECG) were normal. Her past and family history were normal. In addition, she had undergone a renal biopsy about 19 months earlier at a different center, and this was normal with no glomerular or tubulointerstitial pathology. Patchy mesangial immunoglobulin (Ig) A deposits had been noticed. She had not been followed up at that center since she was married and moved to another region of the country. She was not taking any medication. General physical and systemic examinations were normal. Laboratory investigations showed hemoglobin of 8.3 gm/dl with microcytic anemia, blood urea of 30 mg/dl, and serum creatinine of 0.8 mg/dl. Urine analysis revealed 3+ protein with numerous red blood cells per high power field (RBC/HPF) and urine albumin-to-creatinine ratio (ACR) was 412 mg/g. Ultrasound showed normal-sized kidneys with no cysts or hemangiomas. A decision to look for vascular causes for hematuria was taken, and she underwent a CT angiogram of the renal vessels (Fig. 1A–C). The AM angle was < 15° in our patient with severe dilatation noted of the LRV owing to the compressive effect. There was some dilatation of the left gonadal vein, though it was not very engorged. A diagnosis of nutcracker syndrome with LRV compression causing gross hematuria and anemia due to persistent blood loss was made. In consultation with vascular surgery, and considering the longevity of her symptoms and the severity of hematuria, it was decided to proceed with surgery. LRV bypass grafting was done using the left greater saphenous vein, with no intraoperative or postoperative complications (Fig. 1D). The patient received aspirin along with an anticoagulant for 3 months postprocedure. Hematuria subsided gradually over the next 6 weeks, and she is currently asymptomatic.

### Case 2

**Fig. 2:**
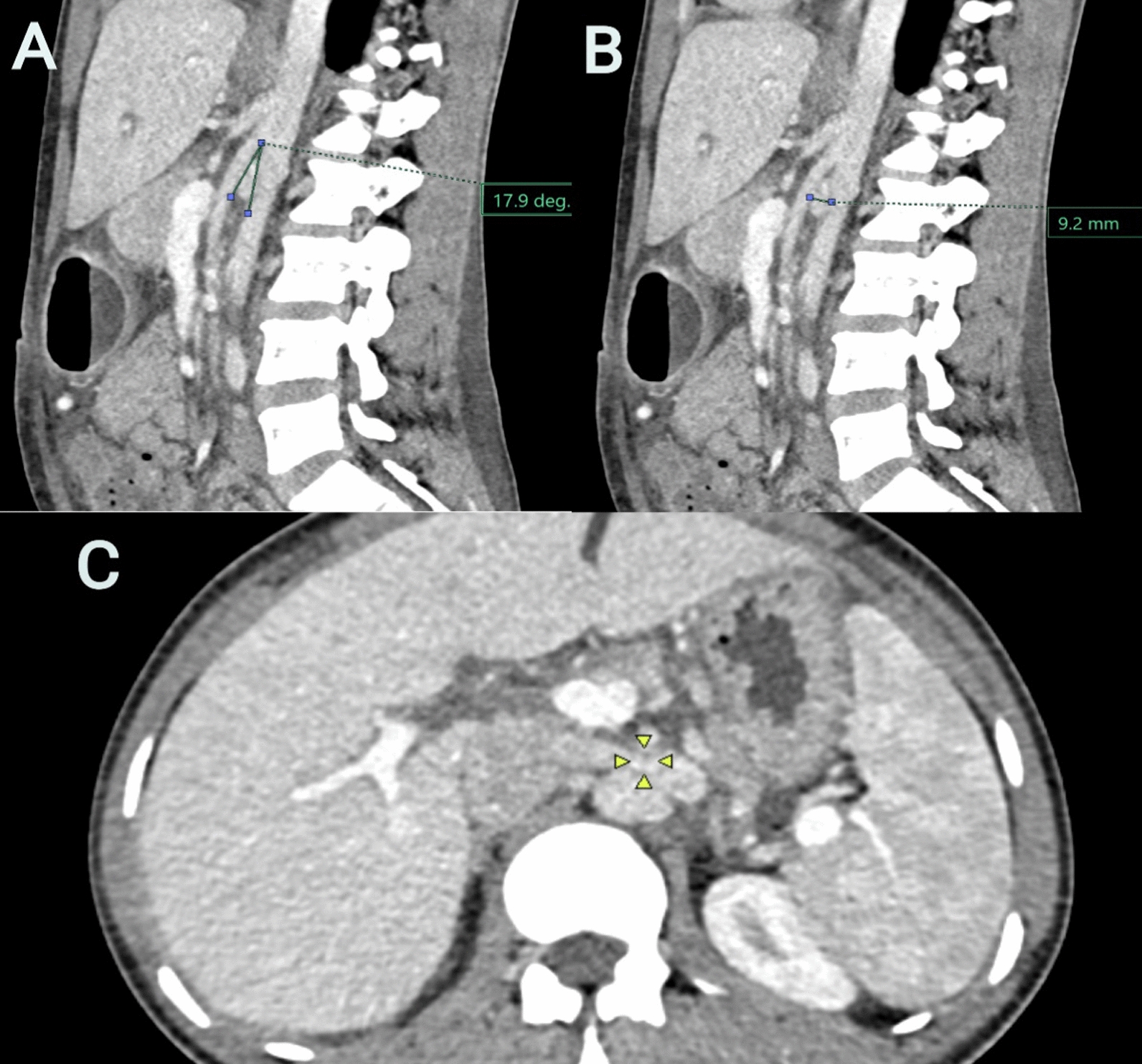
**A** Computed tomography angiogram sagittal section showing an aortomesenteric angle of 17.9 degrees with minimal dilatation of gonadal vein; **B** computed tomography angiogram sagittal section showing an aortomesenteric distance of 9.2 mm; **C** computed tomography angiogram axial section showing dilated renal vein (arrows)

A 30-year-old South Asian female individual presented with a 1-week history of abdominal pain that was insidious in onset, gradually progressive, dull aching, and intermittent in nature. The pain did not have any particular aggravating or relieving factors, and there had been no previous history of similar pain earlier. There was no history of hematuria, weight loss, or fever. She had undergone a tubectomy 7 years earlier. Vitals revealed tachycardia and tachypnea with 132/84 mmHg blood pressure. The abdominal examination did not reveal any localized tenderness, and it revealed normal bowel sounds. Serum hemoglobin was 9.4 gm/dl with microcytic anemia, blood urea was 14 mg/dl, and serum creatinine was 0.7 mg/dl. Urine analysis showed protein 1 + , 6–8 RBC/HPF with 4–5 white blood cells (WBC)/HPF. Ultrasound showed normal-sized kidneys with no cysts or hemangiomas. CT abdomen performed to rule out intraabdominal pathology revealed an AM angle of < 18° in our patient, with severe dilatation noted of the LRV owing to compressive effect. AM distance was > 9 mm (Fig. 2A–C). Following the assessment, the diagnosis of nutcracker syndrome with LRV compression causing abdominal pain was made. She was treated symptomatically with painkillers and discharged with no immediate symptoms. She continues to be on follow-up and has not had any recurrence of her symptoms so far.

## Discussion

The terms nutcracker syndrome and phenomenon are used interchangeably; it has been observed that the anomaly of LRV entrapment is not always associated with clinical symptoms. Table 1 lists the diagnostic criteria based on CT imaging. The term NCS, however, is limited to patients who present with characteristic manifestations such as hematuria (78.5%), left flank pain (40%), pelvic pains, varicocele (35%), proteinuria (30%), and anemia (13.3%) due to persistent bleeding. The lack of definitive diagnostic criteria makes the precise prevalence of NCS remains unknown; however, most patients present at any age, from childhood to around 80 years, with prevalence peaking between 10 and 30 years [5, 6]. It has been postulated that the AM angle decreases as a result of the fast growth of the vertebral bodies and height that happens during adolescence [7]. The two main anatomical variants of NCS are the more common anterior variety and the rarer posterior NCS variety.

**Table 1:** Diagnostic criteria (based on CT imaging)

Serial No	Parameters	Diagnostic criteria for NCS	Case 1	Case 2
1	Aortomesenteric (AM) angle [8]	< 35–39° [definitive diagnosis] (normal value 45–90°) The abrupt narrowing of the LRV between the abdominal aorta and SMA, the characteristic “beak sign” (Fig. 1) in the axial plane, is reported to have an 88.9% specificity and 91.7% sensitivity	12.7°	17.9°
2	Hilar-to-AM diameter ratio	≥ 4.9 (66.7% sensitivity and 100% specificity) [9]	4 mm	9.2 mm
3	Doppler ultrasonography	≥ 3 mmHg indicates venous hypertension [10]	Not performed	Not performed

The pathogenesis of symptoms in NCS typically includes hematuria, left flank pain, and gonadal varices, which manifest as left-sided varicoceles in men and as ovarian vein syndrome characterized by menstrual abnormalities and pelvic pain in women. This last symptom is due to the drainage of the gonadal vein into the left renal vein [11]. Pain is a frequent symptom, which is occasionally linked to the gonadal vein syndrome; the later illness is characterized by flank or stomach pain that may sometimes radiate to the buttock and posteromedial thigh. Varicoceles almost always occur on the left side, occurring in 9.5% of males [12]. Elevated venous pressure in the stenosed LRV is thought to cause varices between the renal pelvis and ureter, venous reflux, and venous hypertension, which can present as micro- or macro-hematuria. Rupture of the thin-walled septum between the renal fornix’s collecting system and small veins can also cause hematuria [13]. It is proposed that, upon standing, venous hypertension within the nephron causes a subclinical immunological cascade in the vascular wall, which may lead to a larger local release of norepinephrine and angiotensin II than necessary [14]. This exaggerated physiologic response to abrupt changes in renal hemodynamics is thought to result in orthostatic proteinuria [15]. The normal pressure gradient between the LRV and the inferior vena cava (IVC) is normally less than 1 mm Hg, but it can rise up to 3 mm Hg or more if the LRV is compressed by the SMA. Few patients suffer severe prolonged symptoms, while others, especially children, remain asymptomatic [16]. Takebayashi *et al*. clinically differentiated NCS into three subtypes: renal bleeding of idiopathic origin, massive orthostatic proteinuria (> 400 mg/day), and severe orthostatic intolerance impairing activities of daily living. Chronic fatigue syndrome has also been associated with NCS with high Left renal vein-Inferior vena cava (LRV-IWC) gradients. Possible coexistence of other conditions such as Henoch–Schönlein purpura, IgA nephropathy, membranous nephropathy, and idiopathic hypercalciuria with urolithiasis concurrent with NCS has been reported.

The treatment of NCS remains controversial and needs to be individualized on the basis of the degree and severity of presenting complaints. In individuals younger than 18 years who are still growing, it is possible that as mesenteric fat reserves increase, the aortomesenteric angle may change, with resolution of changes in 30% of these patients in 24 months [17]. Surgical therapy may be considered for patients with recurrent gross hematuria; anemia; severe flank or abdominal pain; deranged renal function, including persistent orthostatic proteinuria; and varicocele formation; it may be considered for patients in whom conservative management has not shown benefit after 24 months in patients < 18 years of age and after 6 months in adults [18, 19]. The development of venous collaterals over time may help resolve LRV hypertension. Within 24 months, up to three-fourths of younger hematuria patients may no longer have symptoms. Orthostatic proteinuria and renal perfusion have been improved by medical therapy with angiotensin-converting enzyme inhibitors (ACE) inhibitors and aspirin.

Surgical management involves the following two techniques:

1. Open surgery: in 1974, Pastershank operated the first documented case of surgical therapy of NCS by releasing the fibrous entrapment that had compressed the LRV between the AM angle on a 34-year-old man who had flank pain and hematuria [20]. In 2005, according to the study by Hartung *et al*. among 42 cases of NCS treated surgically, 83.3% of patients showed effective symptom remission [21]. Renal autotransplantation and LRV transposition are two recent open vascular techniques. Excision of the veins at the IVC junction and reimplantation distal to the SMA are required for LRV transposition. Despite being a generally low-risk procedure, postoperative complications such as LRV restenosis, small bowel adhesions, and paralytic ileus have all been reported [22]. Robot-assisted or minimally invasive laparoscopic procedures shorten postoperative recovery period and are becoming more popular [23, 24]. Transposition is currently the most often employed surgical technique for NCS and is regarded as the gold standard in management.

Left autologous renocaval bypass [25], as performed in our patient, has been used in a few case reports in the early stages of NCS where gonadal vein distension has not occurred and no other varicosities have been demonstrated. A new venous bypass increases renal blood outflow and relieves LRV hypertension effectively. Long-term results are, however, unknown with this technique.

2. Endovascular treatment is a minimally invasive procedure and has gained success in recent years with advancements in stent technology, with the advantage of quick recuperation and symptom relief. In order to prevent stent thrombosis, postoperative prophylactic antiplatelet and anticoagulant therapy is required for up to 3 months to promote stent endothelization [26]. The stent migration rate was 6.7% according to a study with 75 patients following LRV stenting, with migration occurring to the right heart, IVC, or retrograde to the renal hilum after an average follow-up of 55 months [26]. Endovascular stenting (EVS) is reporting increasing evidence of efficacy, and in centers with skilled surgeons, it can be employed as the recommended treatment strategy. Following the procedure, a regimen consisting of 3 days of low-molecular-weight (LMW) heparin, 30 days of oral clopidogrel, and 3 months of aspirin is used to prevent stent thrombosis and facilitate endothelization [27]. Currently, it is believed that hybrid therapy using a combination of open repair with endovascular stenting combines the strength of both procedures and may help in reducing complications. [28]. Owing to the rarity of nutcracker syndrome, Heilijgers *et al*. [29] recommend that it should be considered in the rare disease registry so that further research is conducted.

## Conclusion

The diagnosis of NCS has to be considered in patients who present with unexplained hematuria or proteinuria. Diagnosis by appropriate imaging studies is necessary, and treatment is dictated by the severity of symptoms. Surgical therapy remains the front-line treatment; however, endovascular techniques are becoming favored owing to advancements in stent technology. Greater understanding is necessary for the optimal management of these patients.

## Data Availability

Not applicable.
